# Spontaneous intracerebral haemorrhage secondary to 5-ALA-induced thrombocytopaenia in a paediatric patient: case report and literature review

**DOI:** 10.1007/s00381-023-05846-y

**Published:** 2023-01-20

**Authors:** Olivia O.T. Mui, Daniel B. Murray, Bill Walsh, Darach W. Crimmins, John D. Caird

**Affiliations:** 1 School of Medicine and Medical Science, University College Dublin, Dublin, Ireland; 2 Royal College of Surgeons in Ireland, Dublin, Ireland; 3 Temple Street Children’s University Hospital, Dublin, Ireland

**Keywords:** Malignant gliomas, Fluorescence-guided surgery, Anaplastic ependymoma, 5-Aminolevulinic acid

## Abstract

**Introduction:**

The primary objective of neurosurgical management of malignant gliomas is maximal safe resection of the tumour. One of the main obstacles in achieving this is the ability to accurately discriminate between tumour edges and the surrounding healthy brain tissue. The use of fluorescence-guided surgery utilising 5-aminolevulinic acid (5-ALA), first introduced more than 20 years ago, has become an invaluable adjunct in high-grade glioma surgery in adults. However, as 5-ALA is not licensed for use in paediatric patients, the safety profile for such use remains undetermined.

**Case report:**

We describe the case of a 4-year-old boy who underwent 5-ALA-guided resection of a fourth ventricle anaplastic ependymoma. Although complete resection was achieved and the patient awoke from surgery well with no neurological deficits, the patient developed acute transaminitis, anaemia, thrombocytopaenia and coagulopathy postoperatively. The patient had a sudden neurological deterioration on postoperative day 2; imaging revealed that he had suffered a spontaneous right frontal intracerebral haemorrhage. The patient returned to theatre for surgical decompression and evacuation of the haematoma, and ultimately went on to make a full recovery.

**Conclusion:**

The use of 5-ALA in paediatric patients can be helpful in maximising surgical resection, but the associated safety profile remains undefined. Further research is urgently warranted in order to characterise the efficacy and risk of the use of 5-ALA in the paediatric population.

## Introduction

Previously, intraoperative visualisation was limited by the surgeon’s ability to identify the tumour’s parameters under conventional white-light microscopy. Preoperative MRI scans can be subject to imprecise registration on the patient’s skin surface anatomy, which can potentially result in inaccurate neuro-navigation intraoperatively. Contrast enhancement on MRI is not always sensitive for tumour margins either.

The development of 5-ALA fluorescence-guided surgery (5-ALA FGS) has become an invaluable neurosurgical advancement in high-grade glioma surgery as it addresses the limitations present with the use of preoperative and even intraoperative MRI. 5-ALA produces fluorescence that allows for real-time identification of tumour borders with high precision, therefore ensuring that a ‘more complete’ resection can be undertaken [1].

5-ALA is naturally produced in humans via the haemoglobin metabolic pathway. When ingested orally prior to surgery, exogenous 5-ALA has remarkable penetration of the blood–brain barrier (BBB) and of the tumour interface. The compound accumulates with high sensitivity and specificity within malignant glial cells before metabolising into the fluorescent metabolite protoporphyrin IX (PpIX) intracellularly. The heightened PpIX production within the tumour cells emit violet-red fluorescence when stimulated by 405-nm-wavelength blue-light microscopy, permitting intraoperative visualisation.

It has been evidenced that 5-ALA use allows for a closer resection to the tumour margins than when relying on preoperative MRI alone [2], and it produces superior results in terms of gross total resection (GTR) and progression-free survival (PFS) [3, 4]. In addition, 5-ALA use is correlated with less residual tumour volumes in postoperative measurements, and patients in this cohort require repeat resections less frequently [3].

### 5-ALA in high-grade glioma surgery

High-grade gliomas (HGGs) are diffuse and infiltrative tumours with poorly defined borders, and neoplastic cells can lie beyond the visible tumour bulk. 5-ALA is proposed to be a sensitive adjunct at delineating tumour parameters; its fluorescence extends beyond the gadolinium contrast-enhancing areas as mapped out on MRI because PpIX is also able to accumulate within the marginal cells [5].

A systematic review and meta-analysis were conducted to investigate the accuracy, extent of resection (EOR) and survival outcomes of the use of 5-ALA. Regarding the diagnostic accuracy of glioblastoma multiforme (GBM) with 5-ALA, overall sensitivity was reported to be 0.87 (95% CI, 0.81–0.92) with specificity of 0.89 (95% CI, 0.79–0.94). Compared to white-light resection, contrast-enhancing tumours were more likely to be completely resected in the patients assigned to 5-ALA, and this cohort demonstrated greater 6-month PFS and overall survival [6].

5-ALA-guided surgery has become an indispensable adjunct in HGG treatment for neurosurgical centres worldwide. Aside from producing consistently successful results and decreasing overall mortality, it is also relatively easy and inexpensive to use. Currently, 5-ALA is only licensed for adults for FGS of malignant gliomas, and it is generally considered safe with minimal adverse effects [7].

### 5-ALA in paediatric neurosurgery

Much the same as in adults, EOR is an important prognostic factor in paediatric HGG surgery. The Children’s Cancer Group HGG study (CCG-945) reported that the 5-year PFS was *double* in those who underwent a surgical resection of 90% or more compared with those who had suboptimal resection regardless of tumour histology [8]. Hence, there is great interest in maximising the likelihood of GTR in paediatric neurosurgery, which could potentially be enhanced by extending the use of 5-ALA beyond the adult population.

Although praised for its superiority over conventional white-light resection of adult glioblastomas, the use of 5-ALA in paediatrics is still unlicensed. The safety profile and presence of any adverse effects is ambiguous; no extensive clinical studies have been performed within this subpopulation. The spectrum of paediatric tumour types is more varied than in adults, and not all are appropriate for FGS with 5-ALA (even if they are intra-axial and contrast-enhancing on MRI) [9]. Aside from malignant gliomas, these tumours include primitive neuroectodermal tumours (PNETs), ependymomas, pilocytic astrocytomas and medulloblastomas, and the value of fluorescence in these cell lineages is uncertain [10, 11]. With a pathobiology distinct from adult tumours [12, 13], the true benefit of using 5-ALA is still unclear alongside concerns of inconsistent fluorescence [10, 14]. For example, Beez et al. showed positive fluorescence in only 50% of paediatric HGGs [15], and Labuschagne found 5-ALA fluorescence to be useful in only 37.5% of cases [10].

The first instance of 5-ALA use in paediatrics was described in 2009 in a patient with pleomorphic xanthoastrocytoma (PXA; histologically a low-grade, WHO grade II, tumour). Intraoperative visualisation was deemed advantageous for successful resection in this case, and aside from transient nausea, the patient did not undergo any complications related to surgery or 5-ALA administration [16].

Since then, several reports have documented successful fluorescence using 5-ALA in paediatrics without any adverse effects [17–20]. In these reports, 5-ALA was found to be effective in achieving a higher rate of GTR in addition to being generally safe to administer to children based on adult dosing guidelines [21].

Malignant gliomas were most likely to fluoresce according to these reports, with patchy and inconsistently non-valuable fluorescence for pilocytic astrocytomas, medulloblastomas and gangliogliomas [9, 21, 22]. This may perhaps further restrict the application of 5-ALA depending on histology. Further investigations are warranted before integration of treatment protocols for these differing tumour types. Nonetheless, it is notable that EOR was still more favourable with suboptimal fluorescence in general, than in cases without [11].

### 5-ALA, hepatotoxicity and coagulopathy

Authors have found a positive correlation between 5-ALA use in adult patients and transient postoperative elevations in liver enzymes (PELE) [23–26]. Although 5-ALA-induced hepatotoxicity appears to be dose dependent, the effects reported were generally temporary and benign; measurements were below threefold the upper limit of normal [23]. Because postoperative liver dysfunction can have many aetiologies and be multifactorial, 5-ALA-induced PELE should be a diagnosis of exclusion especially in adults, where primary liver disease, alcohol or drug use, adiposity and medications commonly affect liver enzymes. Asymptomatic increases in aspartate aminotransferase (AST) and alanine aminotransferase (ALT) can also result from prone positioning, intraoperative hypotension and blood loss. Propofol itself inhibits the cytochrome P450 system, and the resulting accumulation of PpIX alongside its decreased elimination from the hepatobiliary system may result in hepatotoxicity manifesting as PELE as well [25].

That said, PELE has also been observed in neurosurgical patients not administered 5-ALA. It seems that the transient change in enzyme measurements is not strictly indicative of 5-ALA safety; the synergistic effects of invasive surgery, anaesthesia, antibiotics and use of anti-epileptic drugs may all have a role to play. Importantly, no sequelae of liver impairment or evidence of liver failure were reported in these adult patients [27].

A handful of cases have also demonstrated PELE in children [10, 15, 28]. It was frequently the only observed complication attributable to 5-ALA ingestion. None of these patients required further treatment of their hepatic abnormality. Interestingly, greater PELE correlated with younger age [14]. Is there a role for age-related pharmacodynamics in the metabolism of 5-ALA [11]?

Another notable side effect associated with 5-ALA ingestion in adults is post-induction hypotension with a 70% incidence reported in a single-centre retrospective study. The precise mechanism is unclear, but authors have shown that there is a greater risk in female patients, in those with low preoperative baseline blood pressures [29] and in those prescribed with anti-hypertensive medications [23]. A separate systematic review in patients undergoing urogenital surgeries found a 25.5% incidence rate of adverse effects with 5-ALA use, with hypotension accounting for 60% of these [30]. However, all authors have concluded that 5-ALA-associated toxicity was minor, recommending a review of anti-hypertensive and hepatotoxic medications, and to consider perioperative blood pressure monitoring only [23, 26].

A report from one paediatric centre described a fatal complication in one of their patients who underwent 5-ALA-guided resection of a posterior fossa tumour with leptomeningeal disease. She developed a fulminant rash, fever and leukocytosis of approximately 40,000/mL six days postoperatively. Extensive work-up excluded an infectious cause. Liver enzymes, however, were within normal range. The authors concluded that theirs was an exceptional case in the absence of any plausible explanation [22].

A prospective multi-centre study which examined both the efficacy and safety profile of 5-ALA use in adults established a correlation between 5-ALA and haematological abnormalities. These abnormalities, however, were generally found to be mild and self-resolving. These included primary leucocytosis, anaemia and thrombocytopenia. None of the patients included in the study had coagulation or bleeding disorders prior to 5-ALA ingestion, and none developed any long-term haematological disorders following resolution of the perioperative abnormality [26].

Another retrospective single-centre study conducted over 5 years into 5-ALA use in adult brain surgery reported that significant adverse haematological reactions were uncommon when compared to a control group. Haemoglobin and platelets were measured before, immediately postoperatively and at 24 h. No significant differences were demonstrated at any time between the groups. Haemoglobin and platelet counts both remained 20% above baseline at all times. There was no significant difference in transfusion requirements (3.9% in the 5-ALA group compared to 3.8% in the control group). Four patients in the 5-ALA group required reintervention within the first 48 h due to bleeding. However, none of the bleeding complications were associated with thrombocytopenia; all platelet counts were > 150,000/ml. Thus, the authors concluded that side effects of 5-ALA are rare and that changes in haematological indices are likely to be multifactorial, with the role of 5-ALA unclear or even insignificant [31].

## Case report

**Fig. 1:**
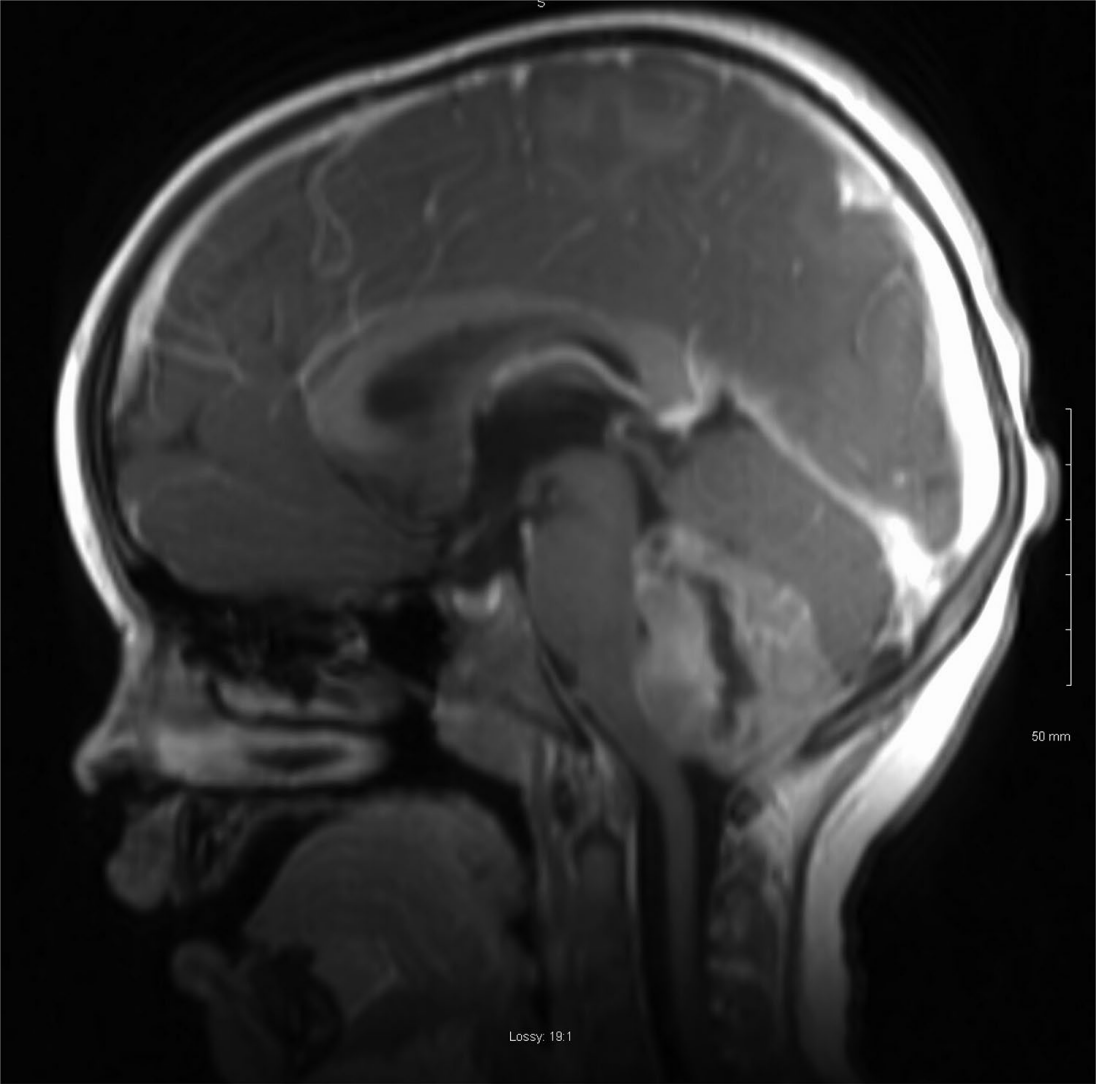
Preoperative MRI brain (sagittal view) of fourth ventricle tumour

**Fig. 2:**
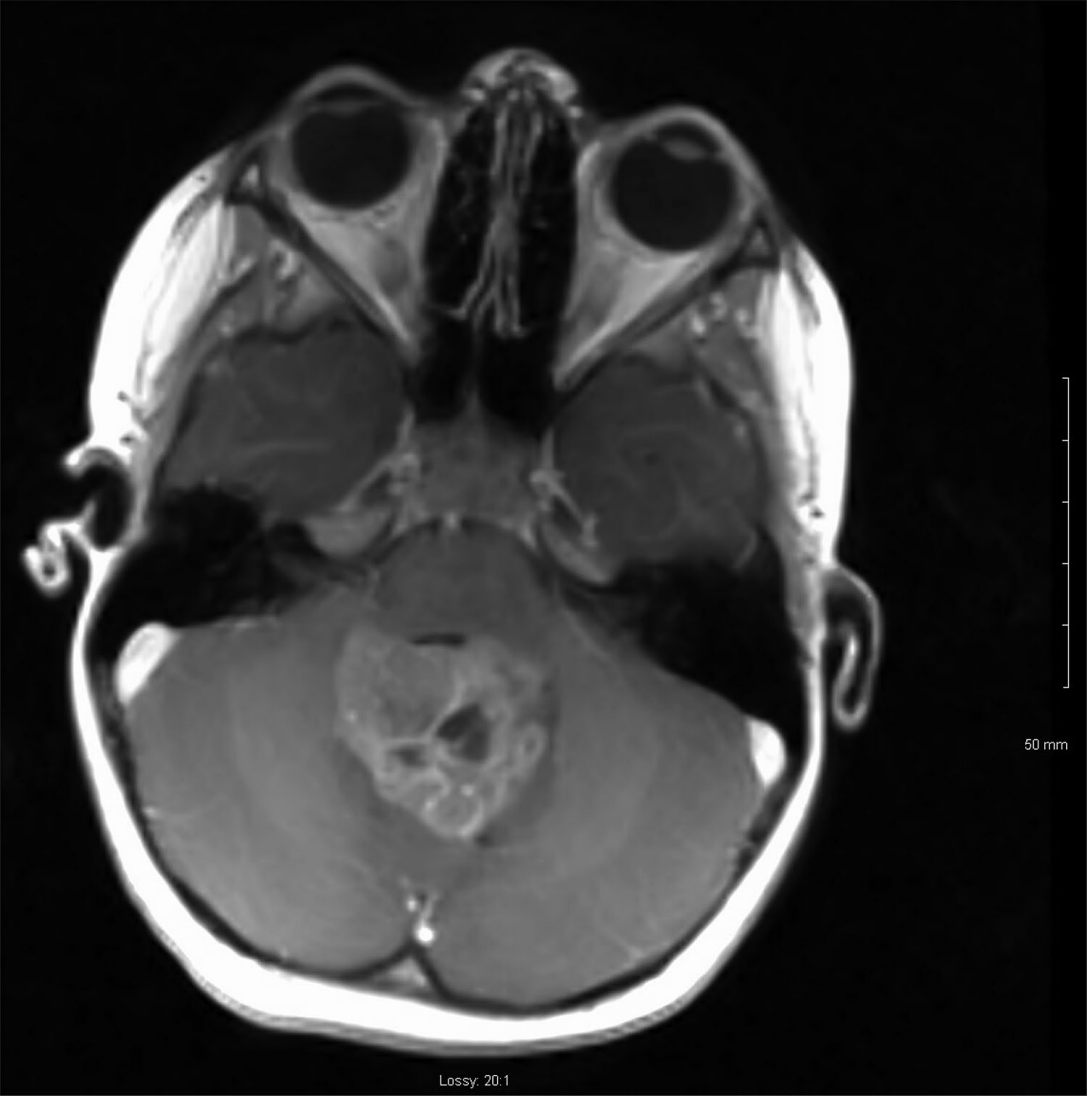
Preoperative MRI brain (axial view) of fourth ventricle tumour

A 4-year-old male was brought to the emergency department with a 2-week history of headache, lethargy and vomiting. CT and MRI brain revealed a 4.5-cm fourth ventricular lesion with associated obstructive hydrocephalus (Figs. 1 and 2). Preoperative bloodwork showed no abnormalities, with normal liver function, platelet count and coagulation profile.

### Surgery

A preoperative dose of 20 mg/kg of 5-ALA (Gliolan) was administered on the morning of surgery (weight 19 kg). The patient underwent insertion of a right frontal external ventricular drain, followed by suboccipital craniotomy, C1 laminectomy and complete resection of the lesion. The tumour fluoresced avidly intraoperatively. There was moderate intraoperative blood volume loss (400 mL); 2 units of red cells and a pool of Octaplex were transfused during the procedure. The patient was positioned prone for a total of 6 h. Immediate postoperative imaging revealed complete radiological resection with no new abnormalities seen. Histological examination of the resected specimen confirmed an anaplastic ependymoma (WHO grade III).

### Postoperative course

The patient was successfully extubated after postoperative MRI on the day of surgery. He remained in the paediatric intensive care unit for monitoring.

Laboratory results on postoperative day 1 revealed a platelet count of 5 × 10^3^/mL with a significant transaminitis (AST 3027 iU/L; ALT 2367 iU/L). The patient was haemodynamically stable and neurologically intact with GCS 15/15.

Despite the liver injury, there was no evidence of acute hepatic failure with PT only modestly elevated (16 s) and no evidence of encephalopathy. Despite treatment with platelets, plasma and red cell transfusions as well as tranexamic acid, vitamin K and fibrinogen, the acute thrombocytopaenia persisted and the patient’s haemoglobin level dropped progressively from 13.5 to 8 g/dL over 36 h. Liver biochemistry results had also worsened with AST peaking at 7198 iU/L and ALT at 4496 iU/L.

**Fig. 3:**
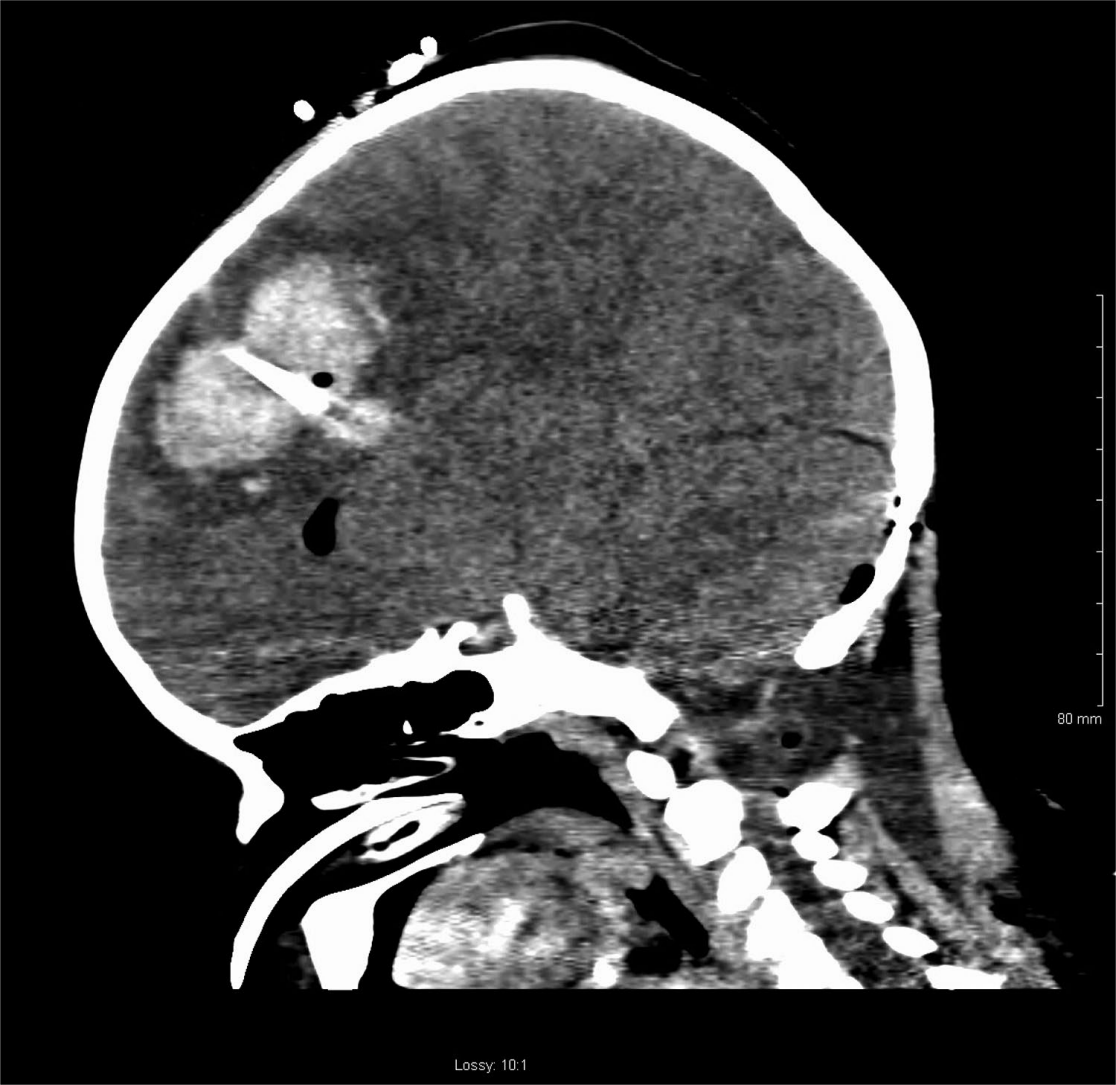
Postoperative CT brain (sagittal view) of spontaneous intraparenchymal haemorrhage along right frontal EVD tract

**Fig. 4:**
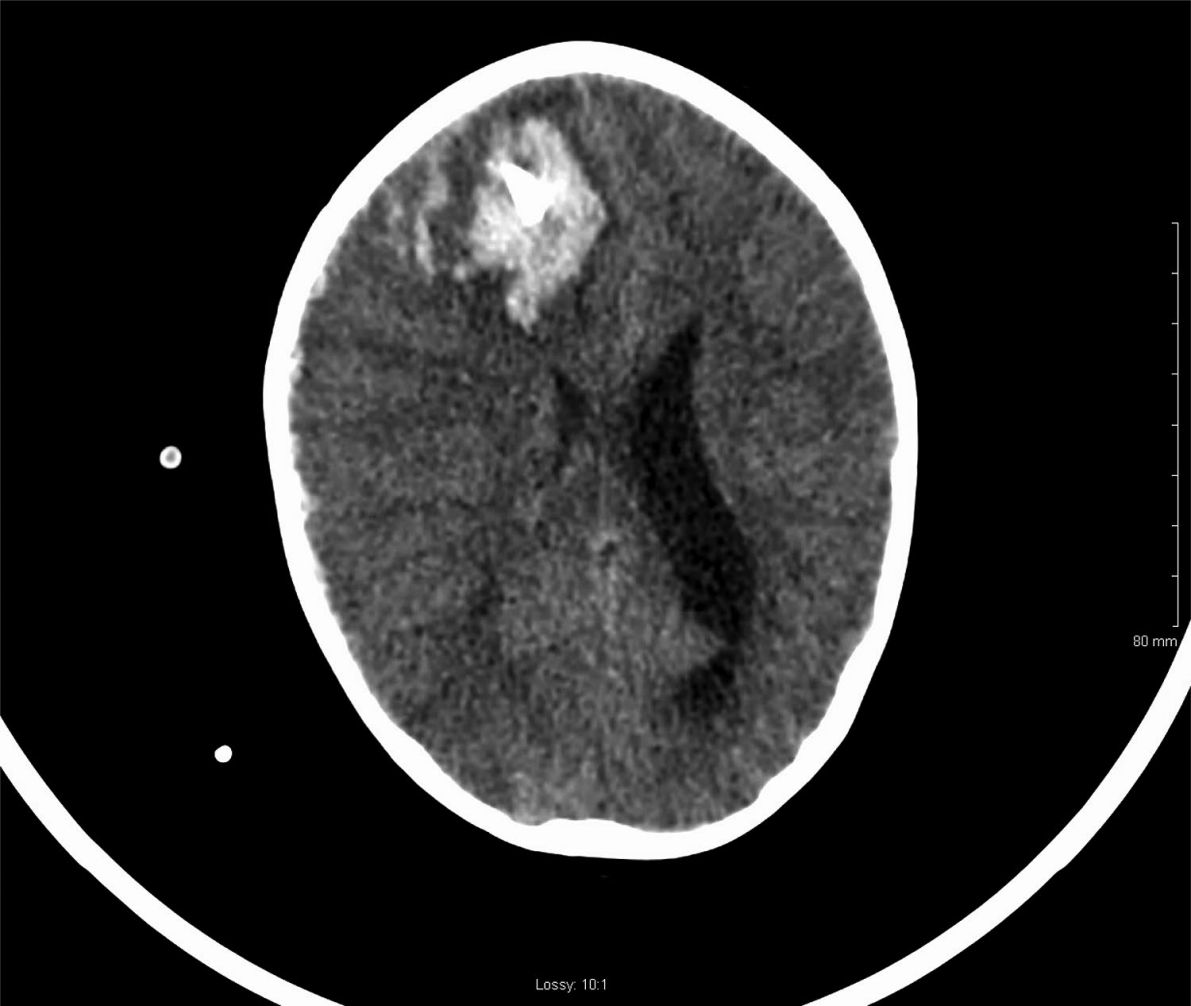
Postoperative CT brain (axial view) of spontaneous frontal haemorrhage

On postoperative day 2, the patient’s external ventricular drain became blocked with blood clots, and he became drowsy. The right pupil became dilated and non-reactive. CT brain showed a new spontaneous right frontal intraparenchymal haemorrhage with intraventricular extension as well as acute subdural haematoma (Figs. 3 and 4). The patient was brought to theatre for emergency craniectomy and evacuation of the haemorrhage. Intraoperative bleeding was difficult to control, and the massive transfusion protocol was enacted. Eventually, once haemostasis was achieved, the patient was brought back to PICU for ongoing management. The transaminitis improved over the following days and the thrombocytopaenia also resolved.

Autologous cranioplasty was performed 1 month later. The patient then received adjuvant proton-beam therapy and chemotherapy. Three months later at outpatient follow-up, the patient was noted to be neurologically intact aside from a very subtle weakness of his left hand. Blood results were all within normal range. He was attending school full-time once again and had made an almost complete clinical recovery.

## Discussion

There is no doubt that 5-ALA plays an important role in optimising surgical treatment of malignant brain tumours. The safety profile of 5-ALA use in adult patients has been reassuring with few reports of significant adverse events reported. The safety profile in paediatric patients, however, is unclear. As the product is not licensed for use in this population, data is scant.

It has been shown that transient transaminitis can occur with 5-ALA use and that the effect is more profound in paediatric patients [14]. Our patient had a significant increase in ALT and AST within hours of the administration of 5-ALA, followed by a sharp decline in platelet count. This resulted in a spontaneous intraparenchymal haemorrhage 48 h postoperatively, necessitating emergency surgical evacuation.

Following extensive multidisciplinary involvement, it was felt that the administration of 5-ALA was the most likely precipitative factor for the thrombocytopaenia. Despite the transaminitis, synthetic liver function was not greatly impaired, and the liver injury was not thought to be responsible for the decrease in platelets. The extent of the liver injury was not significant enough to cause that degree of thrombocytopaenia, and the degree of transaminitis was also disproportionate to the liver injury.

Acute liver failure is defined as INR > 1.5 *and* encephalopathy. Our patient displayed no evidence of encephalopathy, although INR was 1.6. Bilirubin was only modestly increased (23 μmol/L). Another marker of liver synthetic function, albumin, was normal. Furthermore, no haematological cause was found. Although we are aware of anecdotal reports of other 5-ALA-related haemorrhages in the paediatric population, there have been no such cases documented in the literature.

It may also be possible that the phenomenon observed in our patient could be partially explained by mitochondrial dysfunction. Anderson et al. reported a case of a 47-year-old patient who developed severe lactic acidosis intraoperatively during 5-ALA-assisted glioma resection. The patient was subsequently managed medically and did not develop any long-term complications [32]. The authors question whether the accumulation of the metabolite PpIX might have been responsible for inducing an acute porphyria-like state during which mild transaminitis is common [33]. However, acidosis is not a recognised complication of acute porphyria, and arterial blood gases (ABGs) play no role in their diagnosis. In the absence of any evidence, or indeed other reports of a similar nature, this seems to be another exceptional case.

Our patient had no personal or family history of coagulation disorders. The differential diagnosis for the life-threatening haemorrhage includes hepatic injury secondary to the long operative time in prone position with moderate intraoperative blood loss. In the absence of any other end-organ injury (i.e. renal, cardiac), we posit that 5-ALA was the most likely causative factor. We thus recommend that controlled 5-ALA safety and efficacy trials be carried out in the paediatric population.

## Conclusion

5-ALA-guided resection of gliomas enables greater accuracy when defining the border between pathological and normal brain tissue, thus increasing the extent of resection and improving rates of progression-free survival. This report summarises the case of 5-ALA-induced transaminitis and thrombocytopaenia in a 4-year-old patient that resulted in spontaneous intracerebral haemorrhage.

Despite having an established safety profile in adult patients, the use of 5-ALA in paediatric patients remains off-label only, and there is a shortage of relevant clinical data available for this reason. The authors thus recommend that a controlled clinical trial of 5-ALA in the paediatric population should be performed, with a specific focus on safety. We do note that a European multicentre trial is currently ongoing which is evaluating the effectiveness and clinical safety profile of 5-ALA in children and adolescents, and we await these results eagerly.
